# Supplementary material for: In vitro modeling of Batrachochytrium dendrobatidis infection of the amphibian skin
Source: PLoS One. 2019 Nov 14;14(11):e0225224. doi: 10.1371/journal.pone.0225224 (PMC6855447; doi:10.1371/journal.pone.0225224)

Negative control 4 hours sham infection

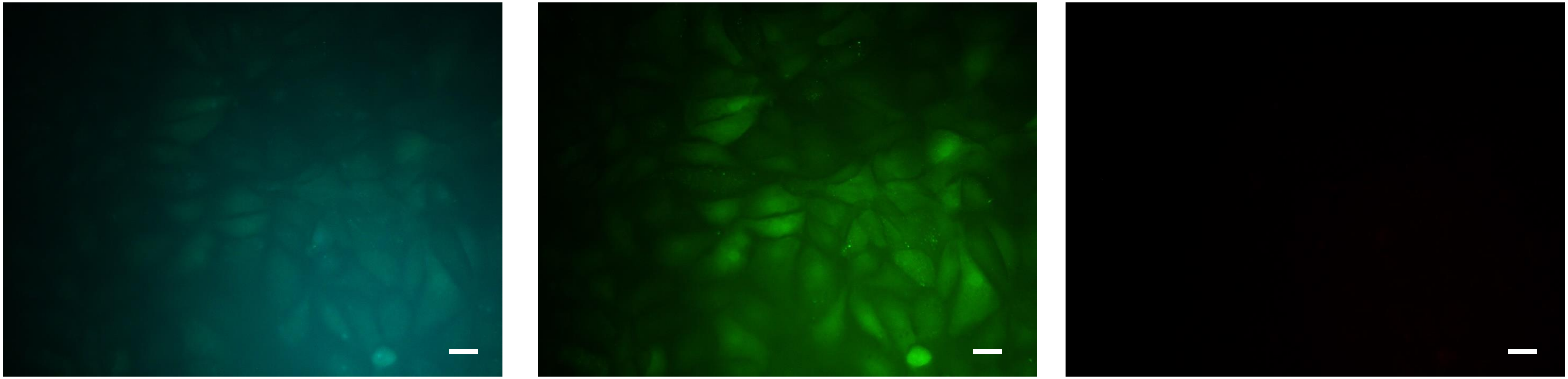

Negative control day 1 sham infection

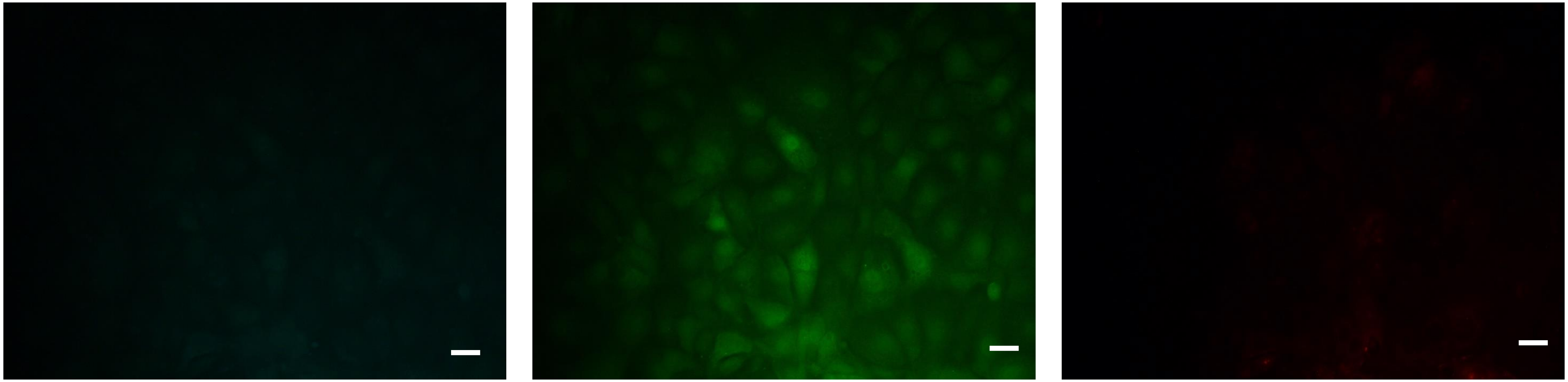

Negative control day 2 sham infection

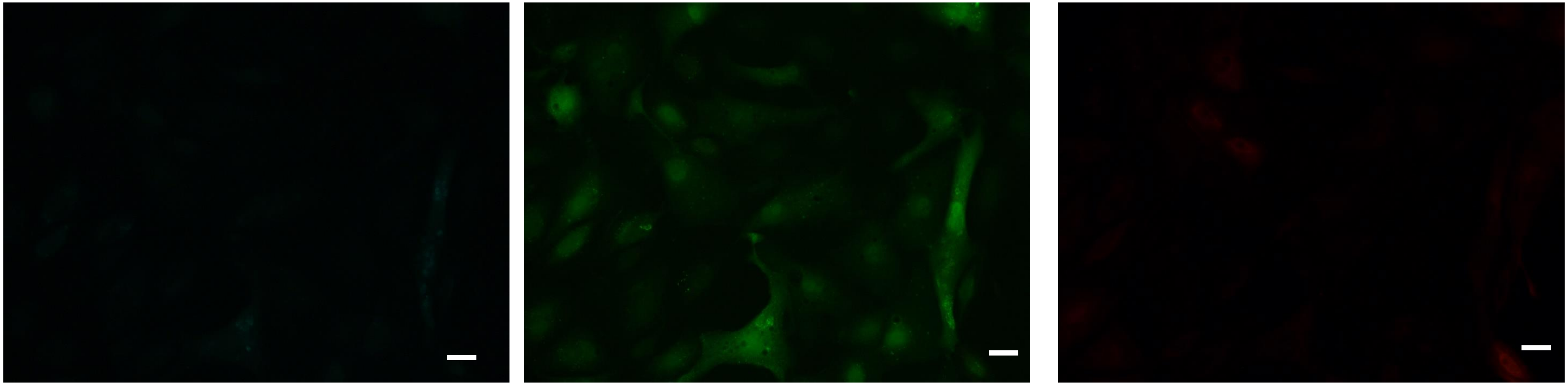

Negative control day 3 sham infection

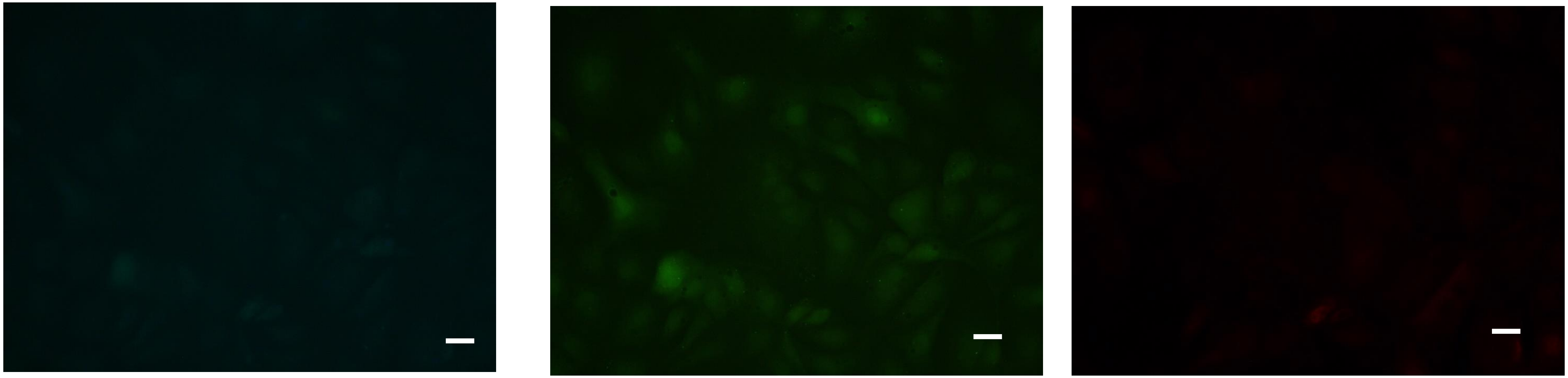

Negative control day 4 sham infection

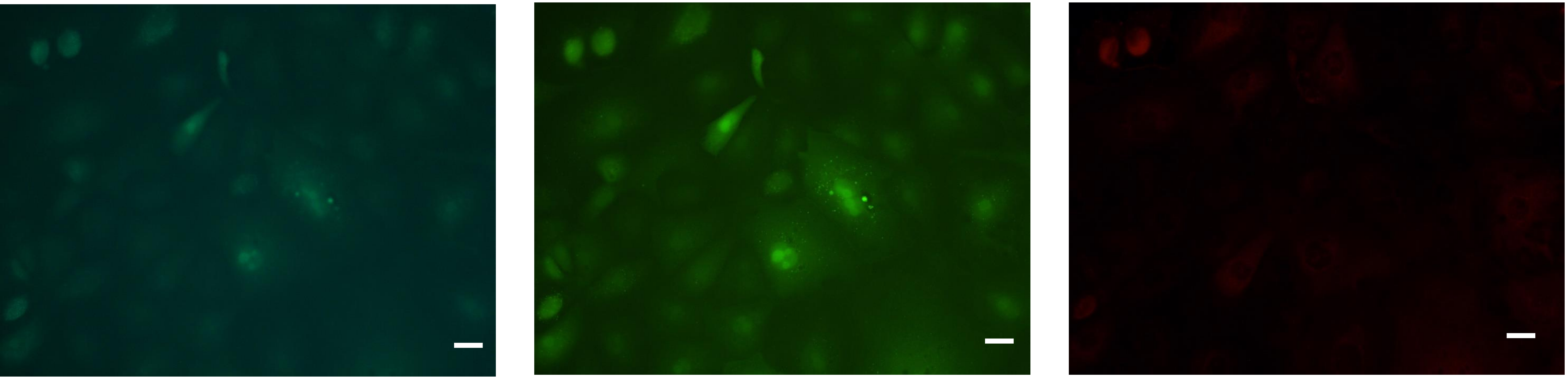

Negative control day 5 sham infection

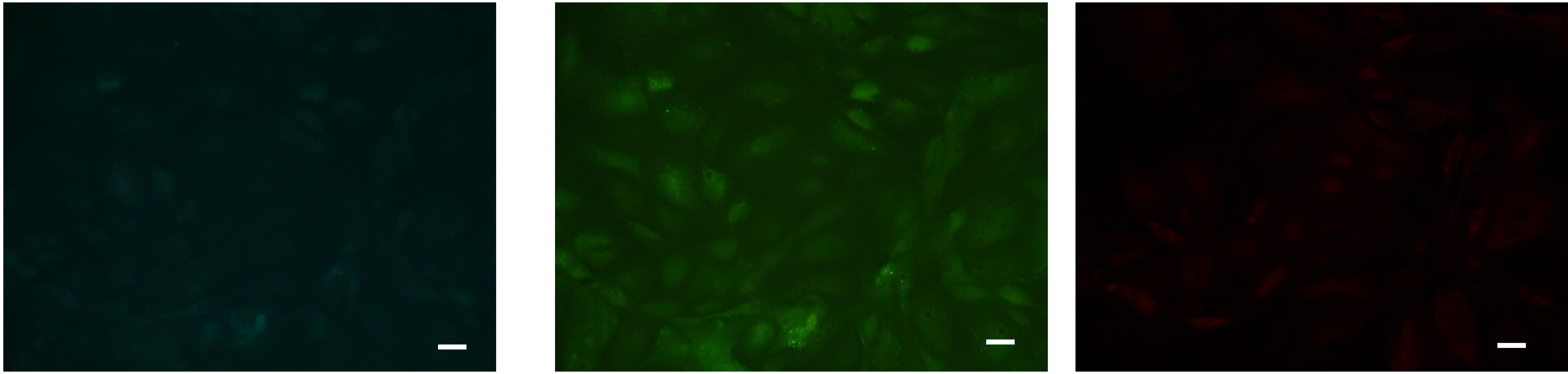

Negative control day 6 sham infection

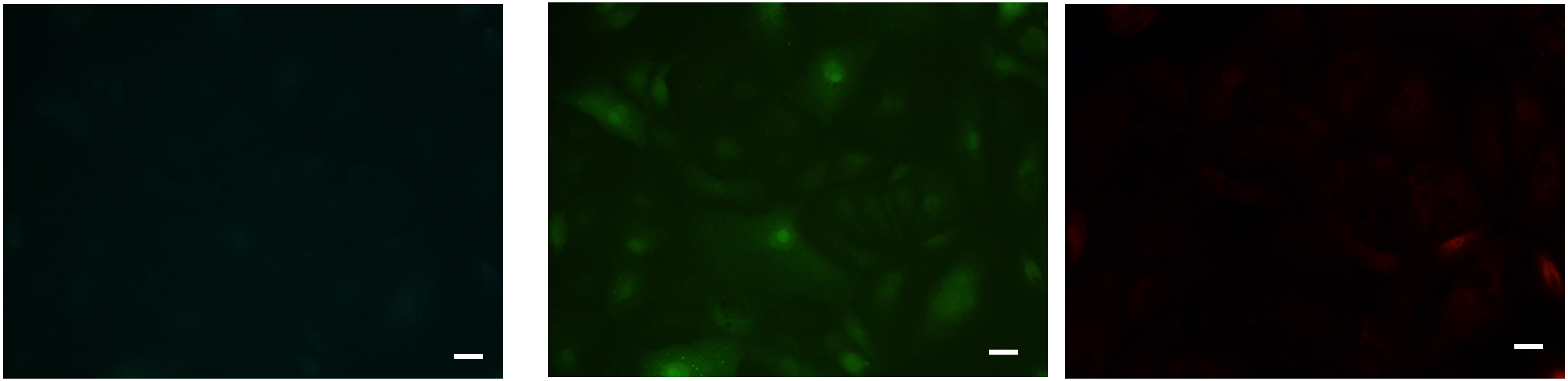

Supplement: S1 File — Shown are the individual fluorescent signals of Bd-infected A6 cells (green cell tracker), extracellular Bd (Calcofluor White (blue)) and extra-and intracellular Bd (Alexa Fluor 568 (red)) of A6 cells at different time points after sham-infection (4 hours to 6 days). Scale bar = 20 μm. (PDF) [file pone.0225224.s001.pdf]
