# Supplementary figures and images for: In vitro modeling of Batrachochytrium dendrobatidis infection of the amphibian skin
Source: PLoS One. 2019 Nov 14;14(11):e0225224. doi: 10.1371/journal.pone.0225224 (PMC6855447; doi:10.1371/journal.pone.0225224)

**Figure 3H**

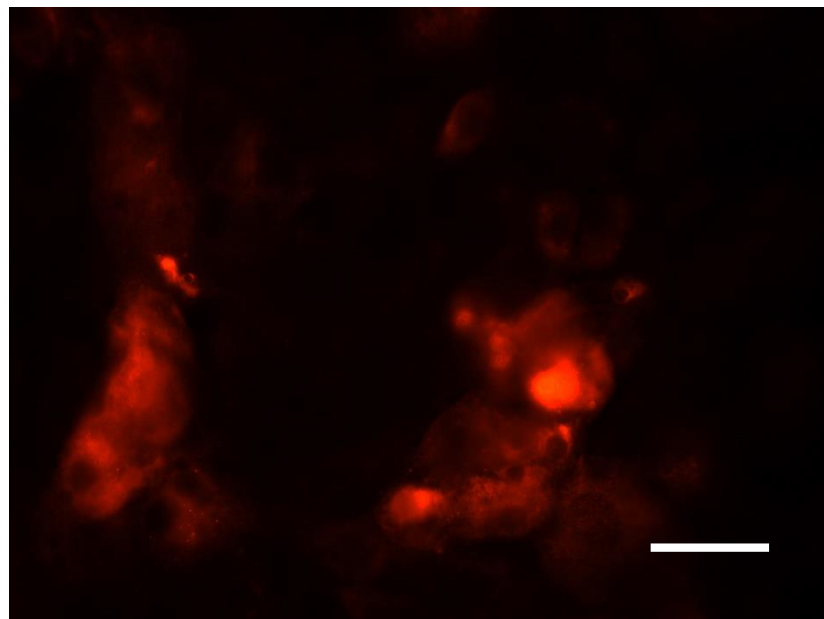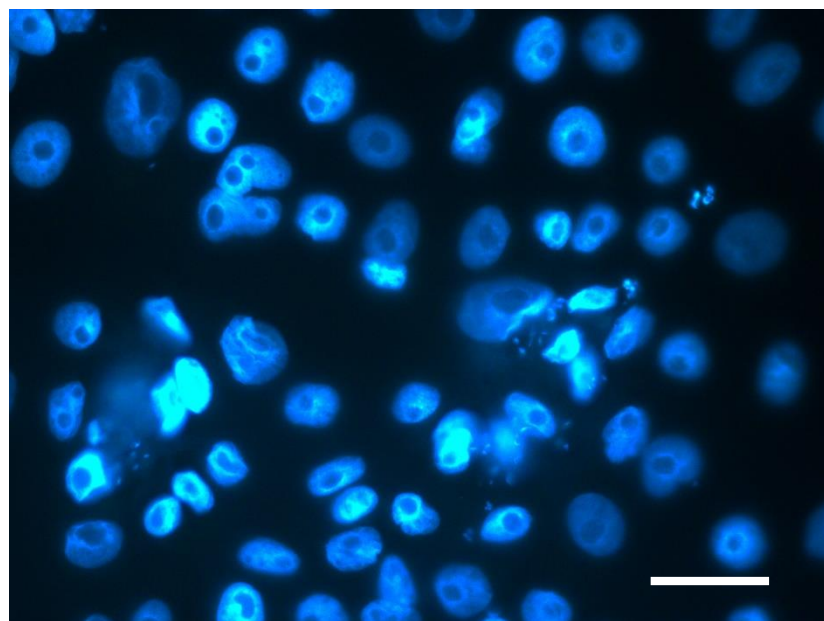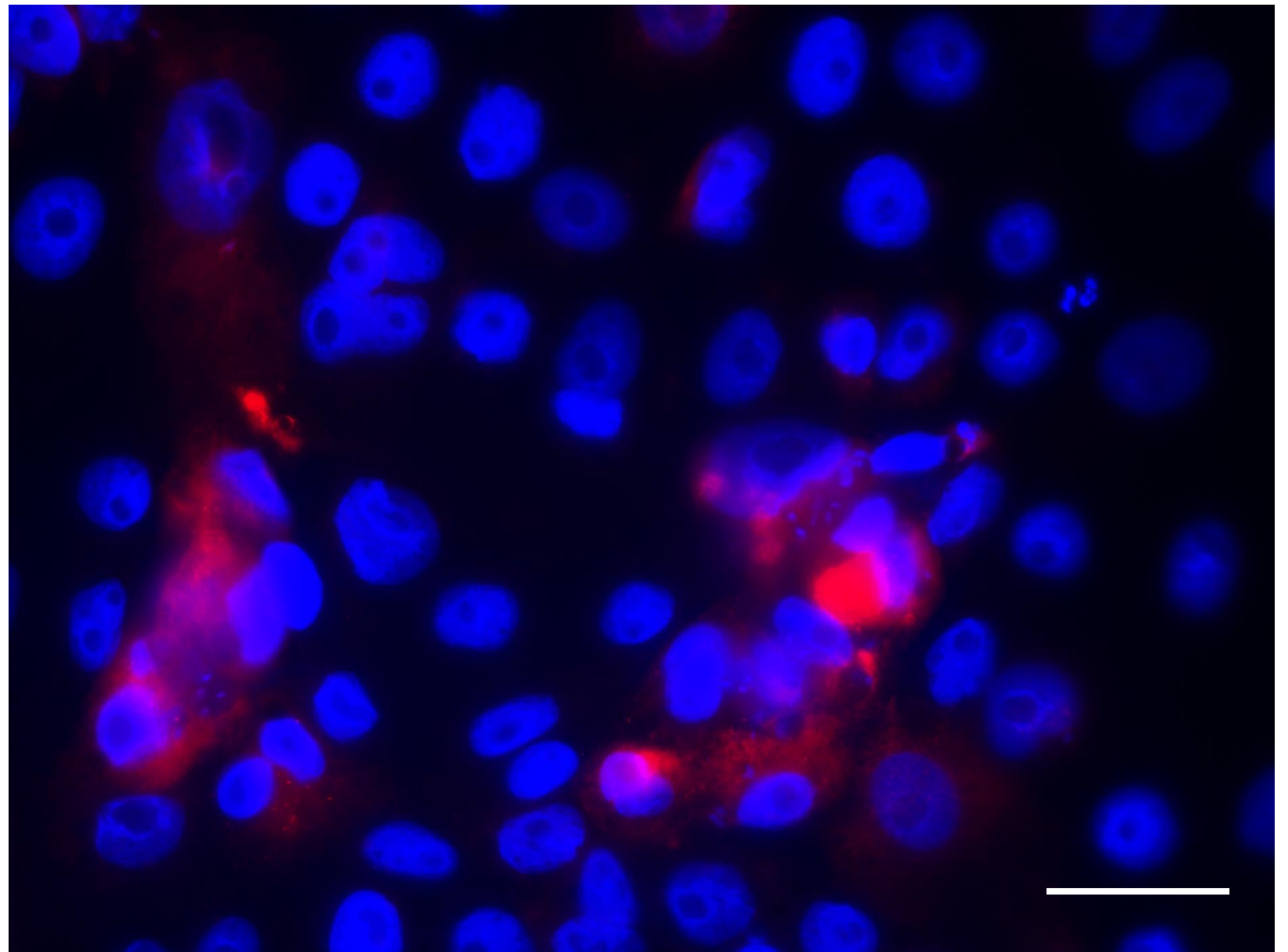

## Negative control day 5 sham infection

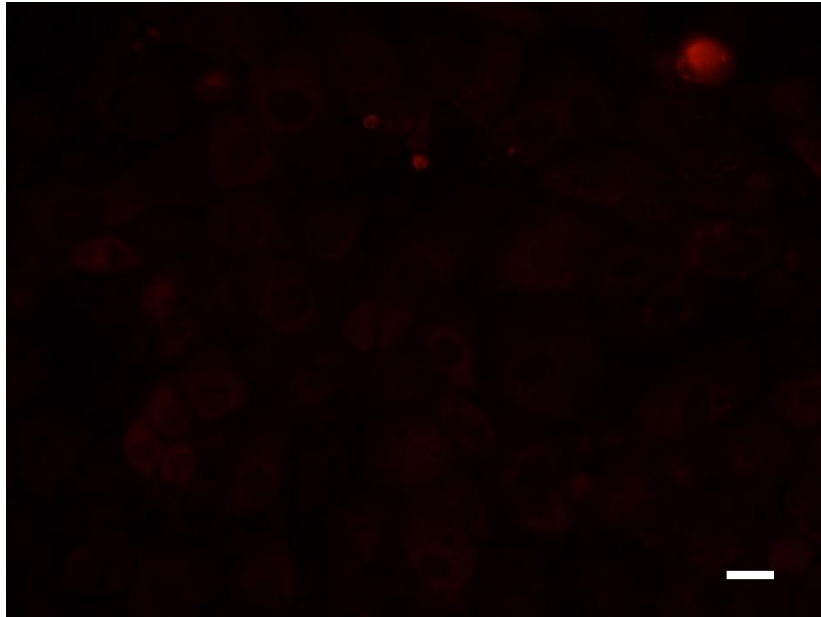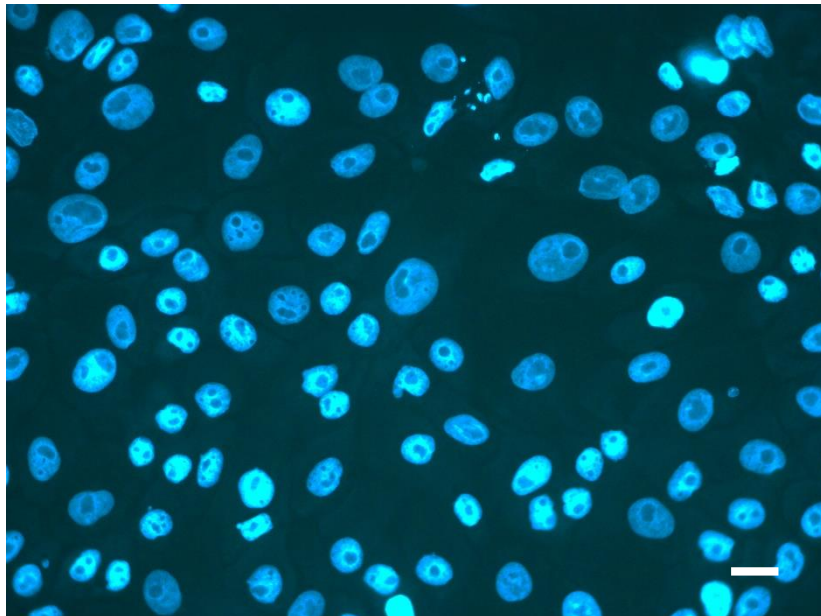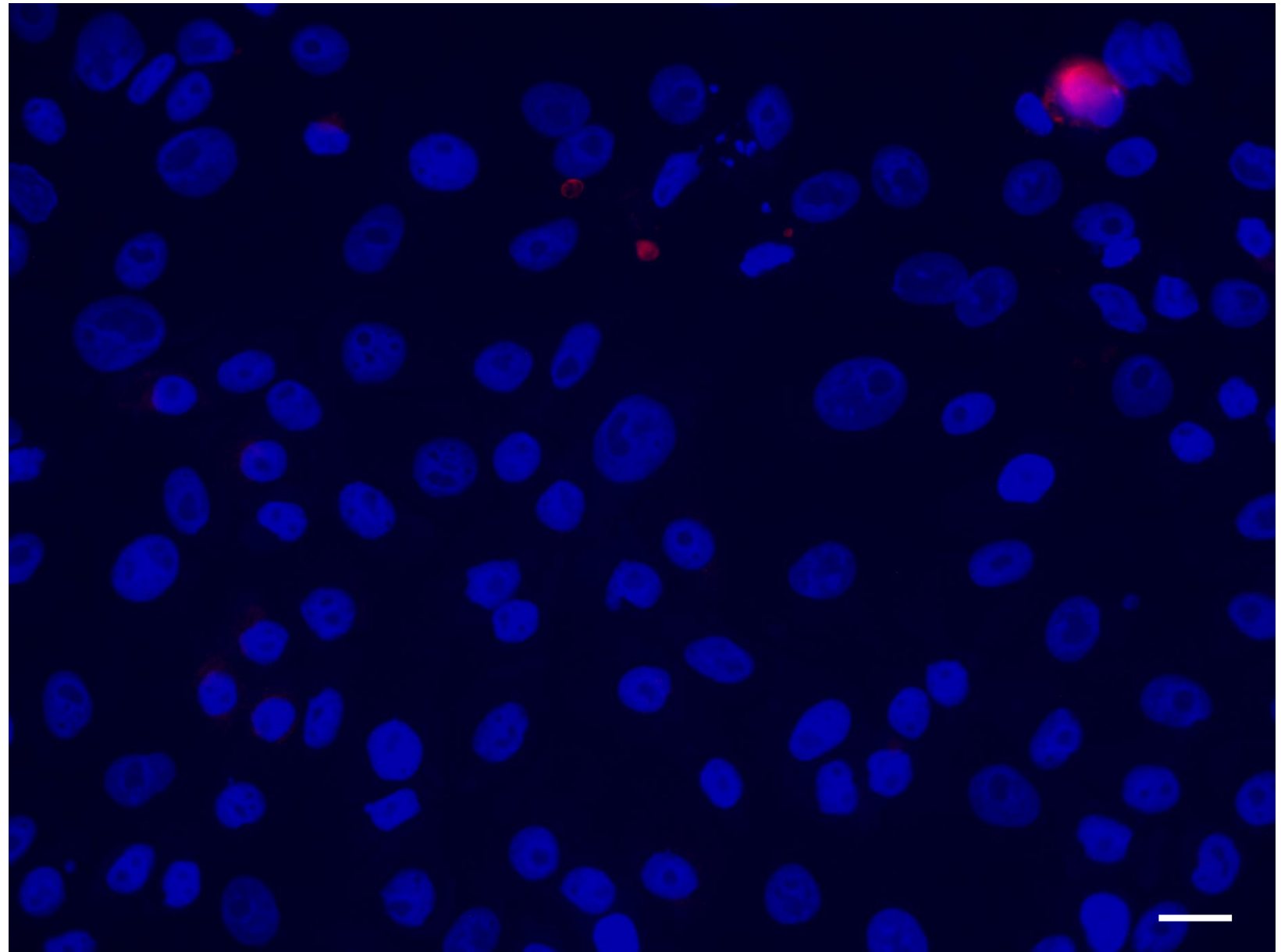

## Positive control day 5 sham infection

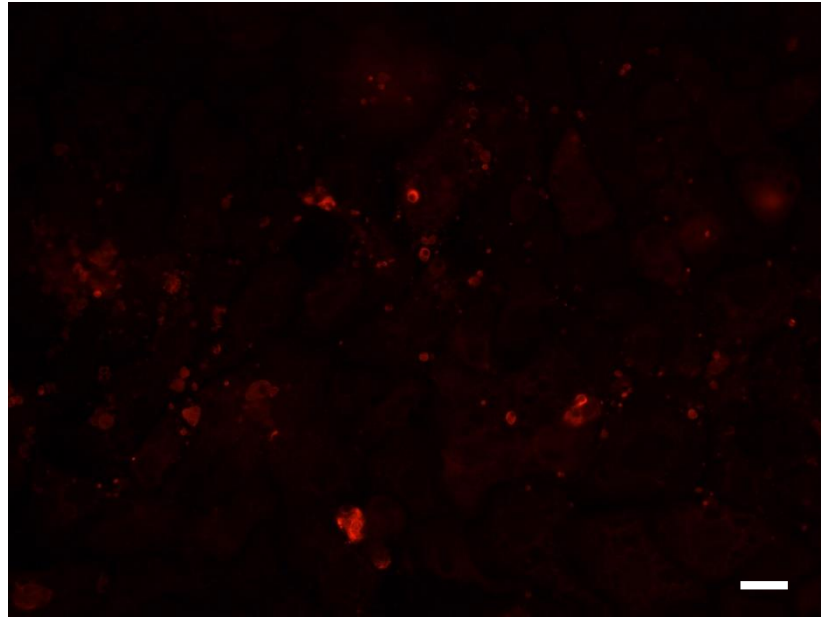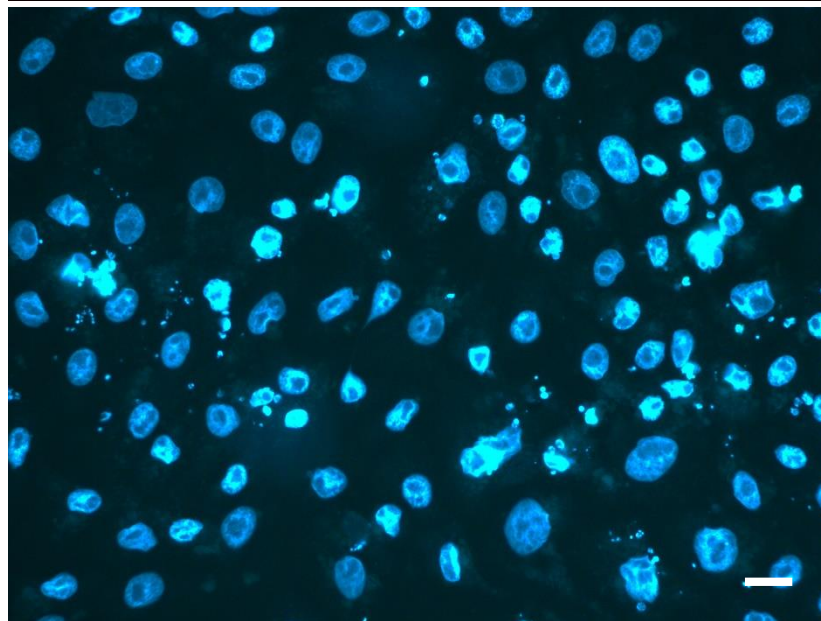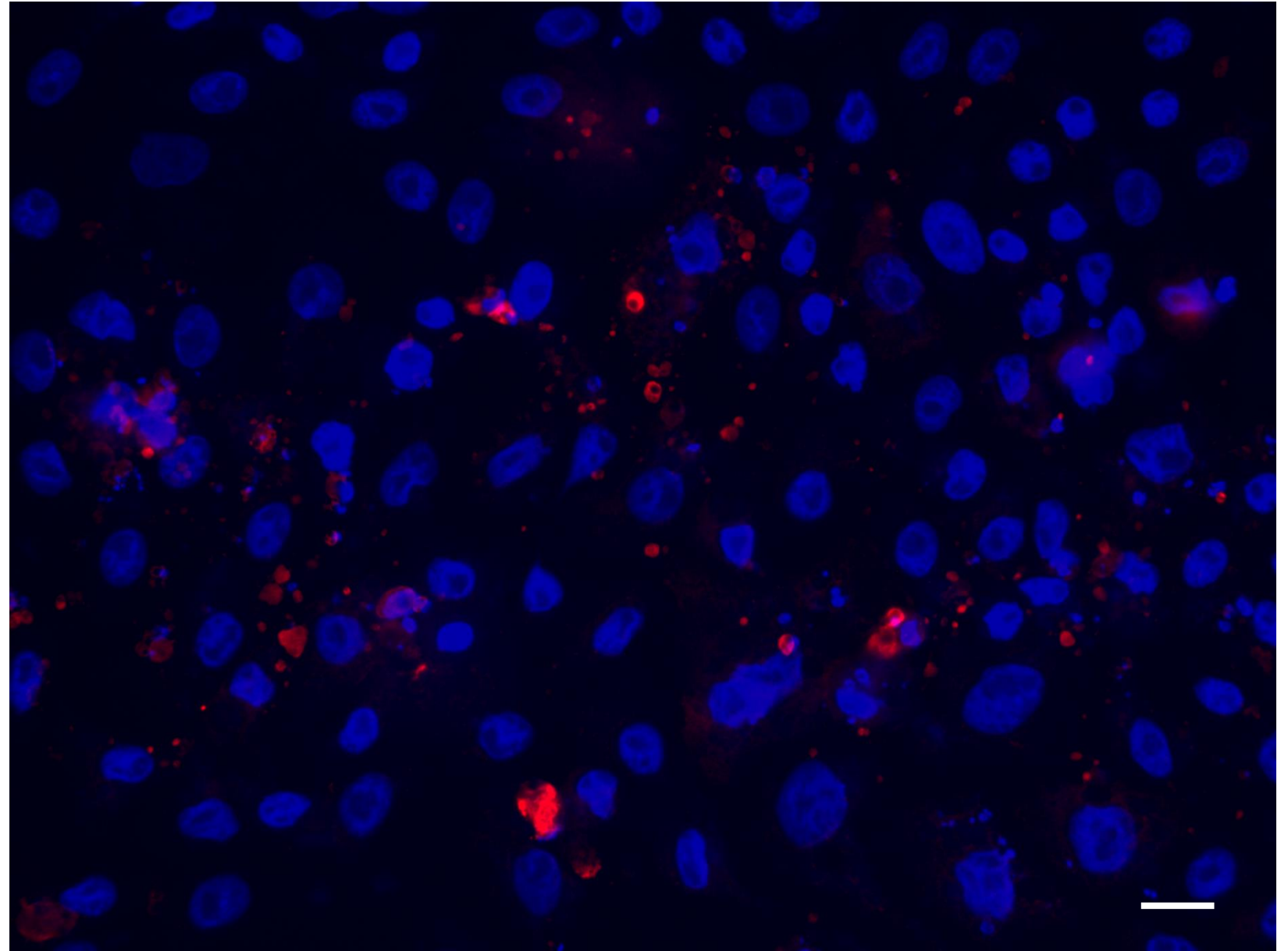

Supplement: S2 File — Shown are the individual fluorescent signals of caspase-3 activation (Alexa Fluor 568 (red)) and nuclear content (Hoechst (blue)), which were used in Fig 3. As a negative control sham-infected A6 cells were included and staurosporin-treated A6 cells (1 μM; 24 hours) served a positive control. Scale bar = 20 μm. (PDF) [file pone.0225224.s002.pdf]

**Figure 3A**

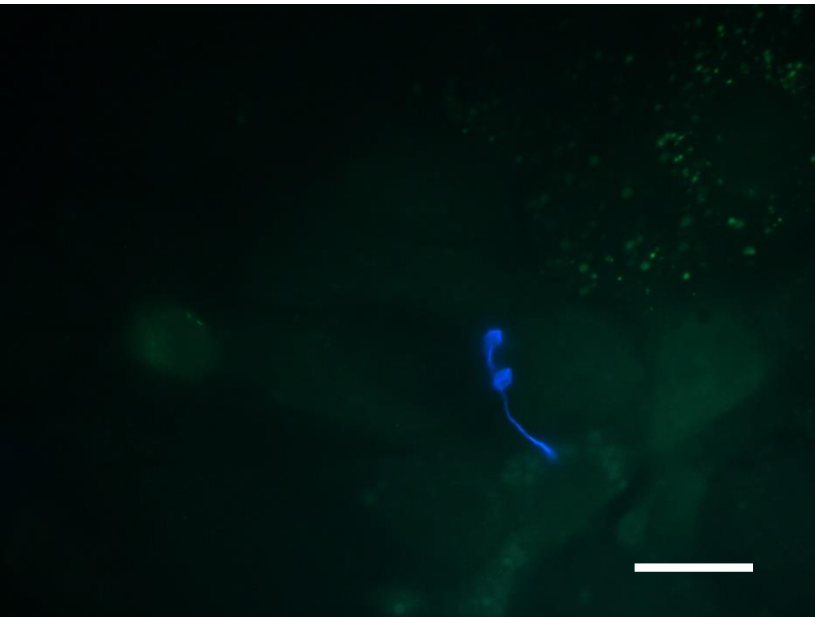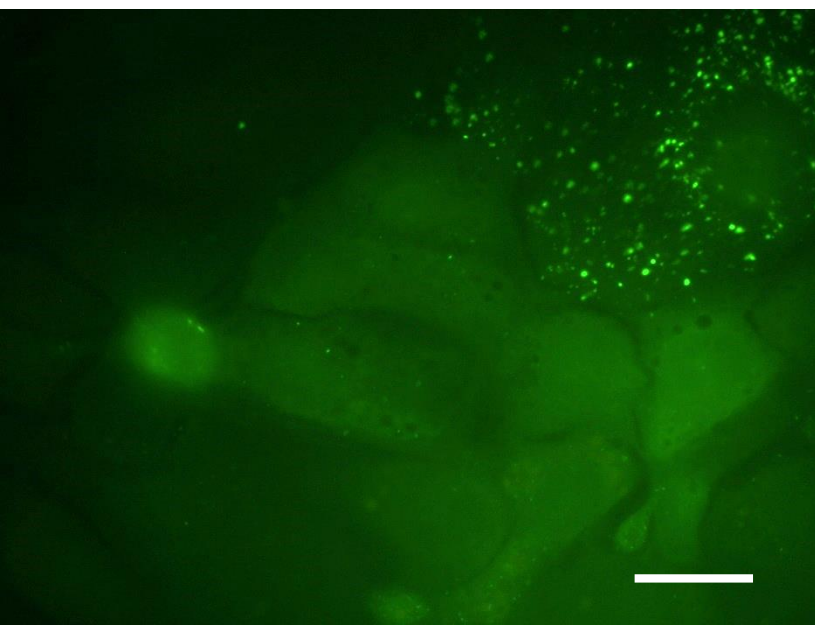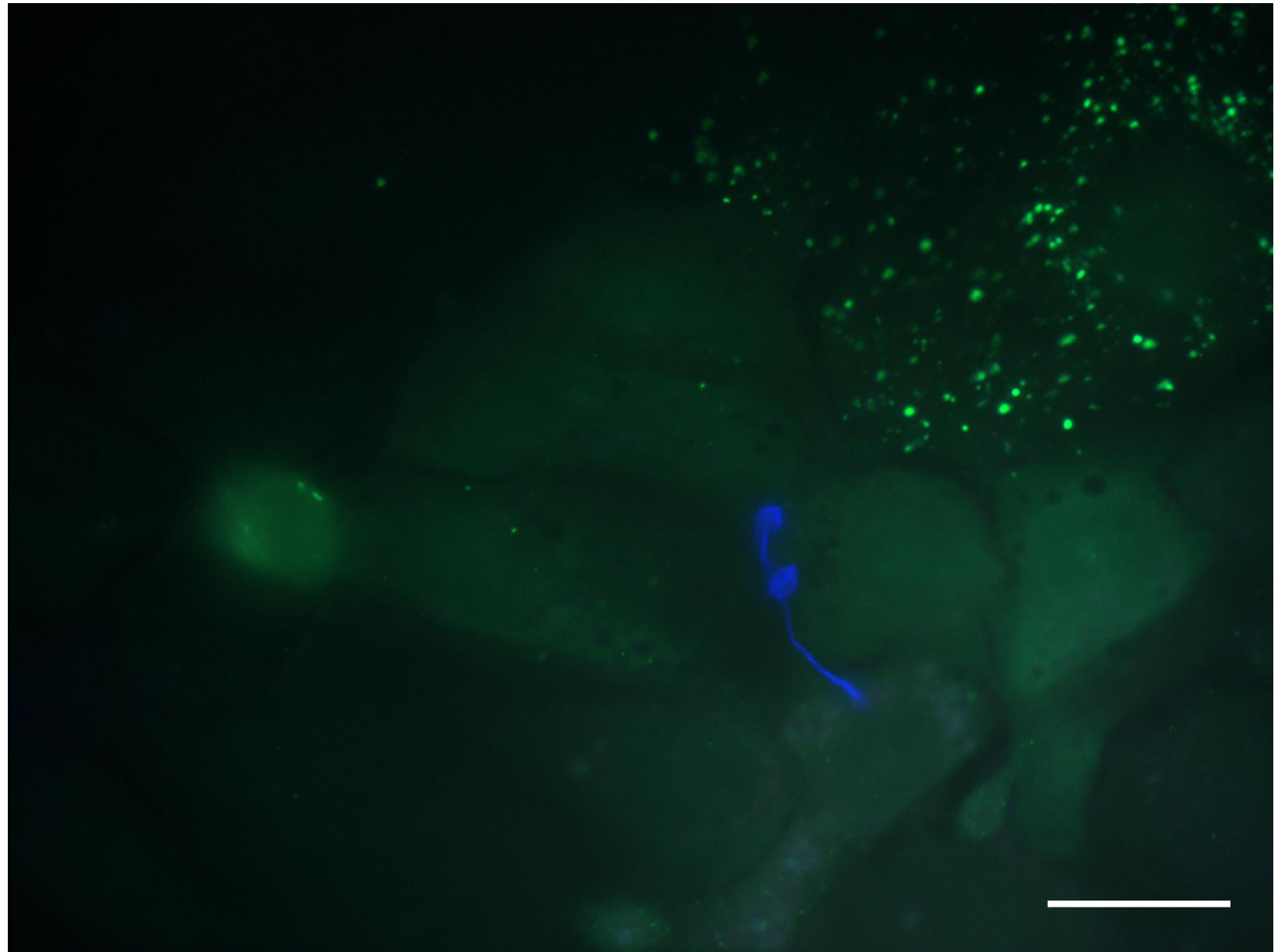

**Figure 3B**

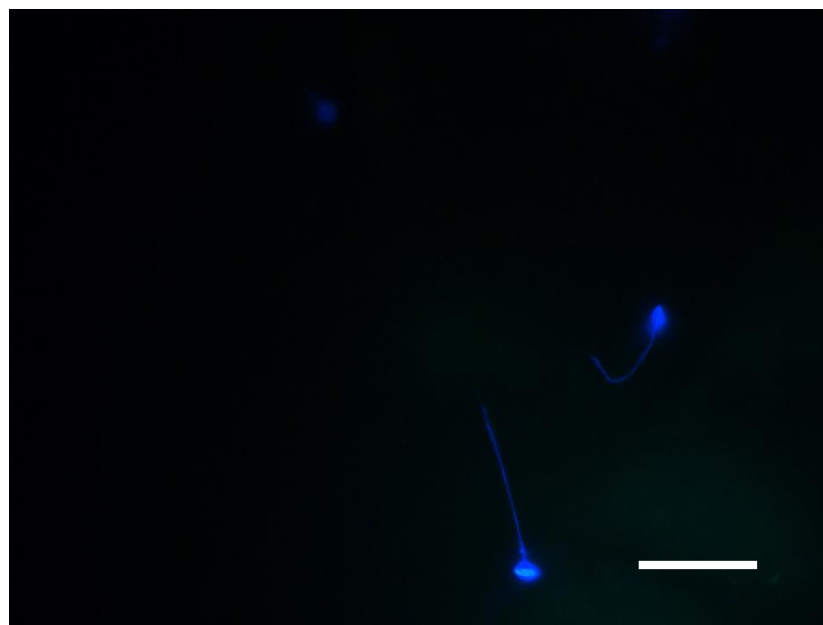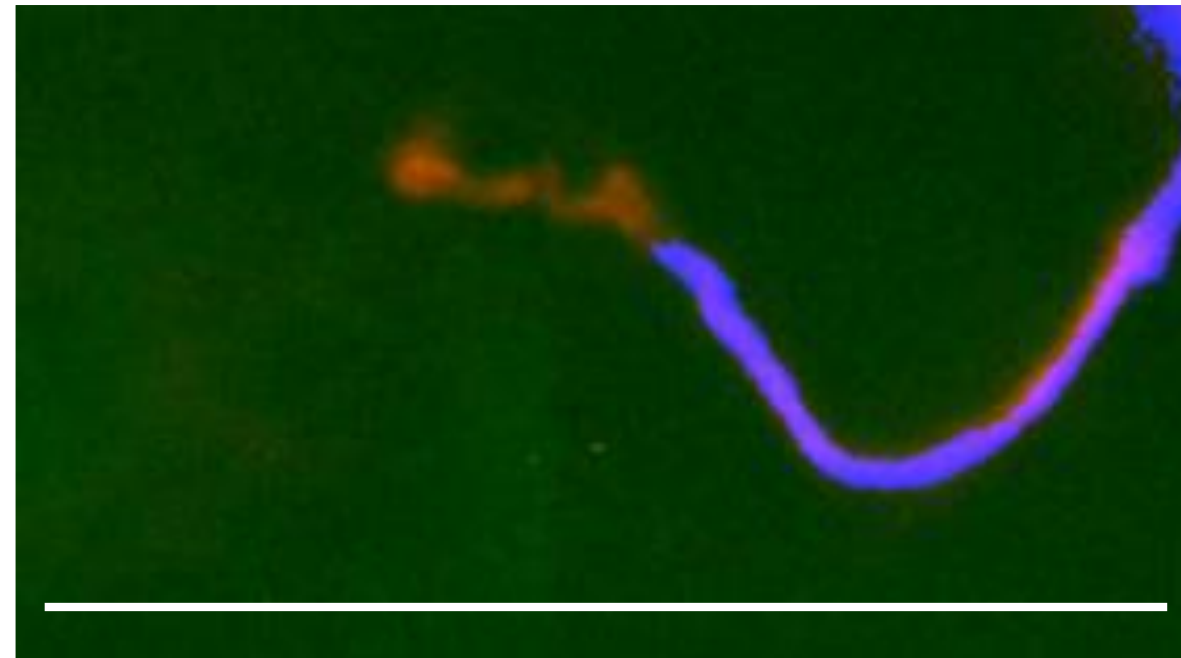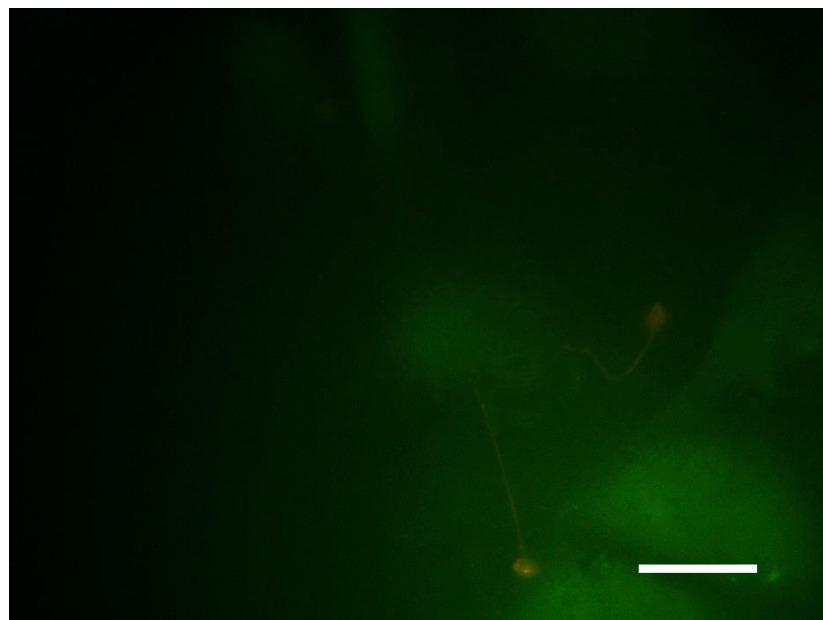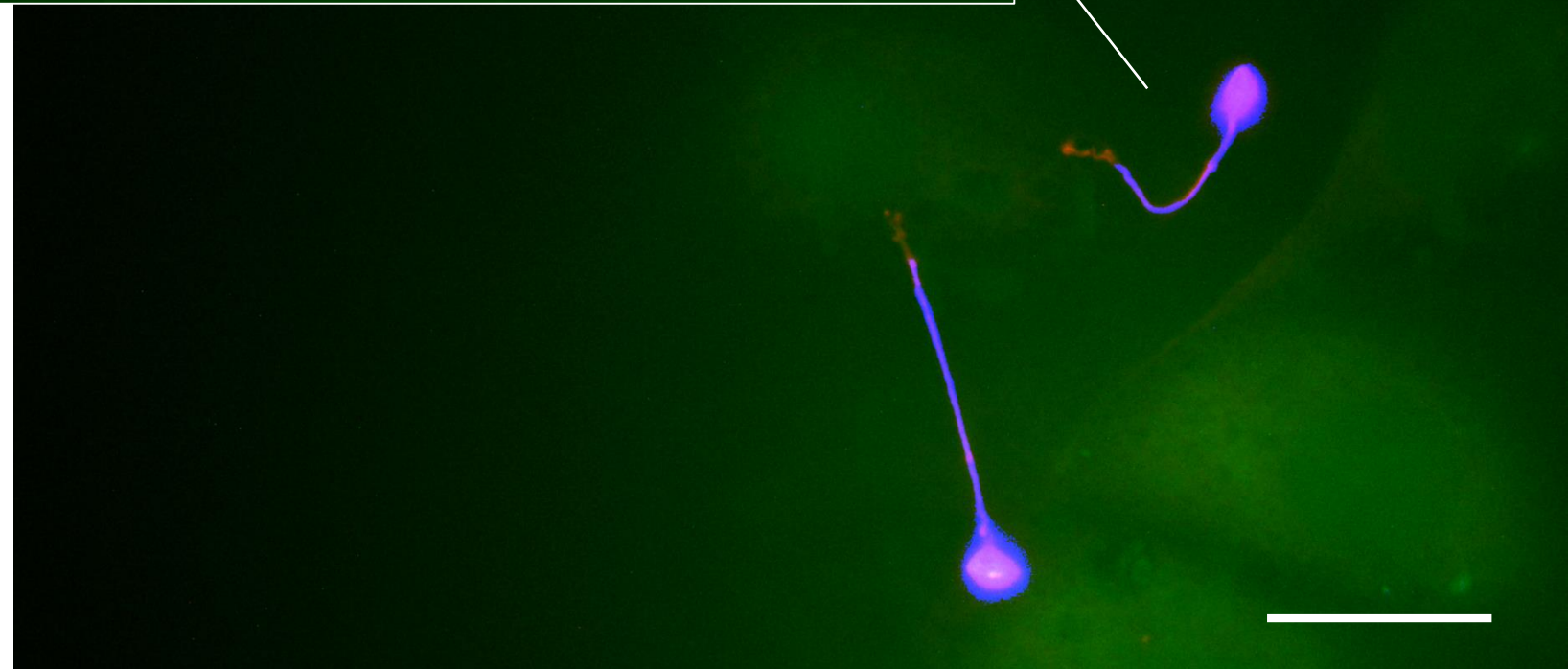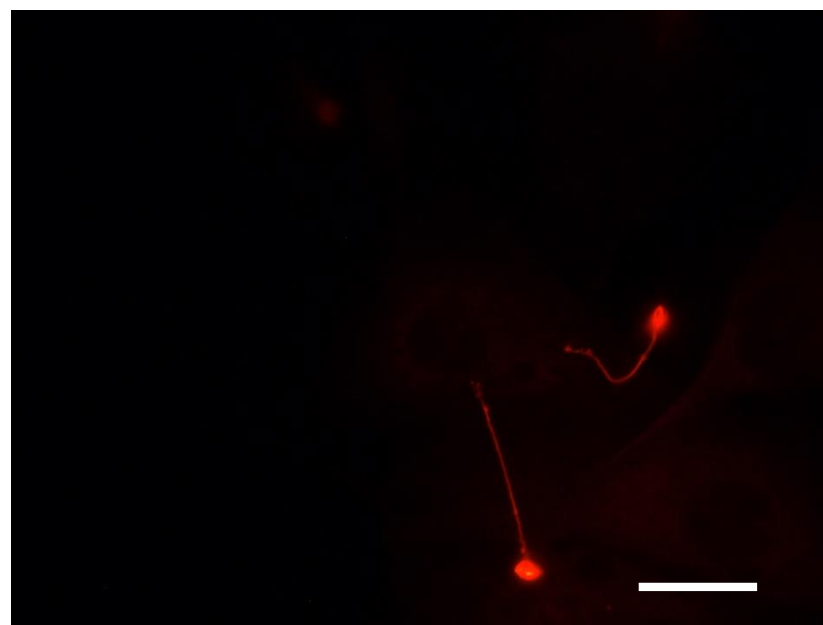

**Figure 3C**

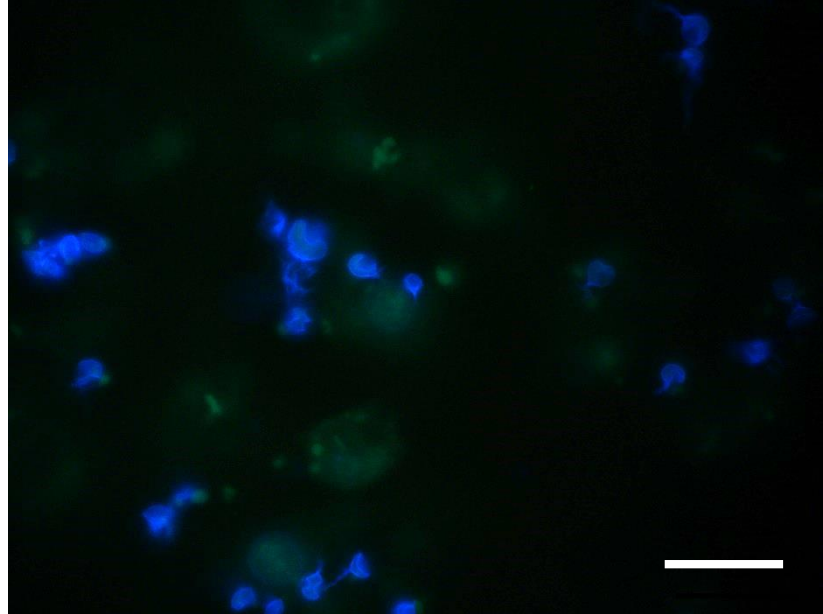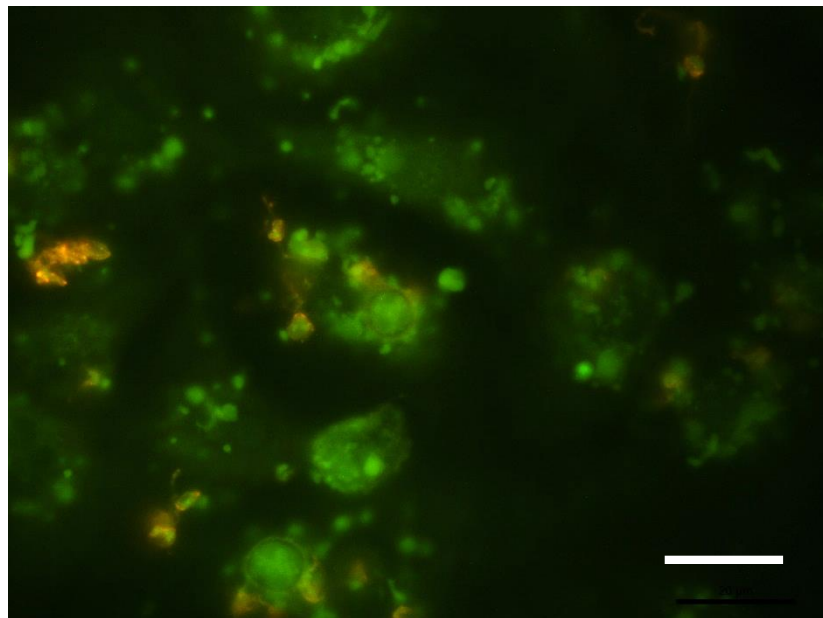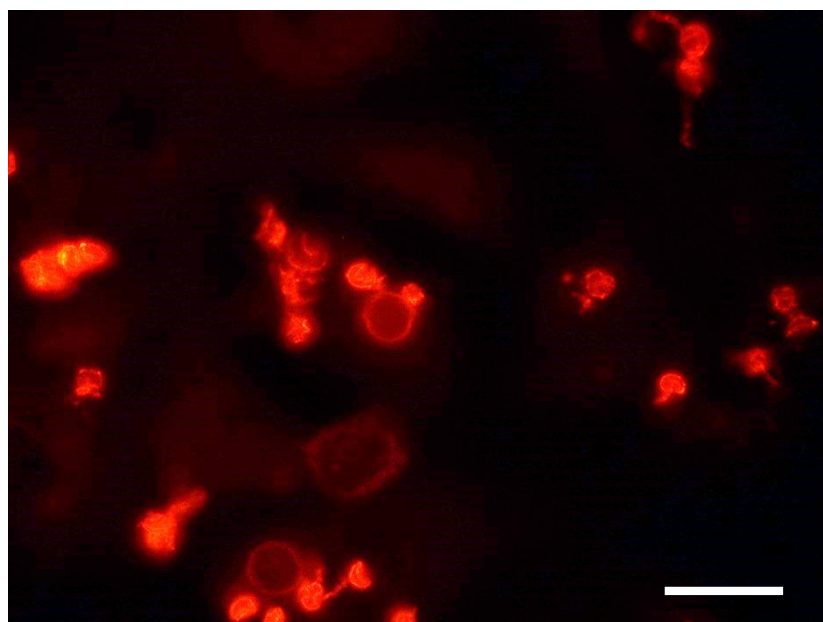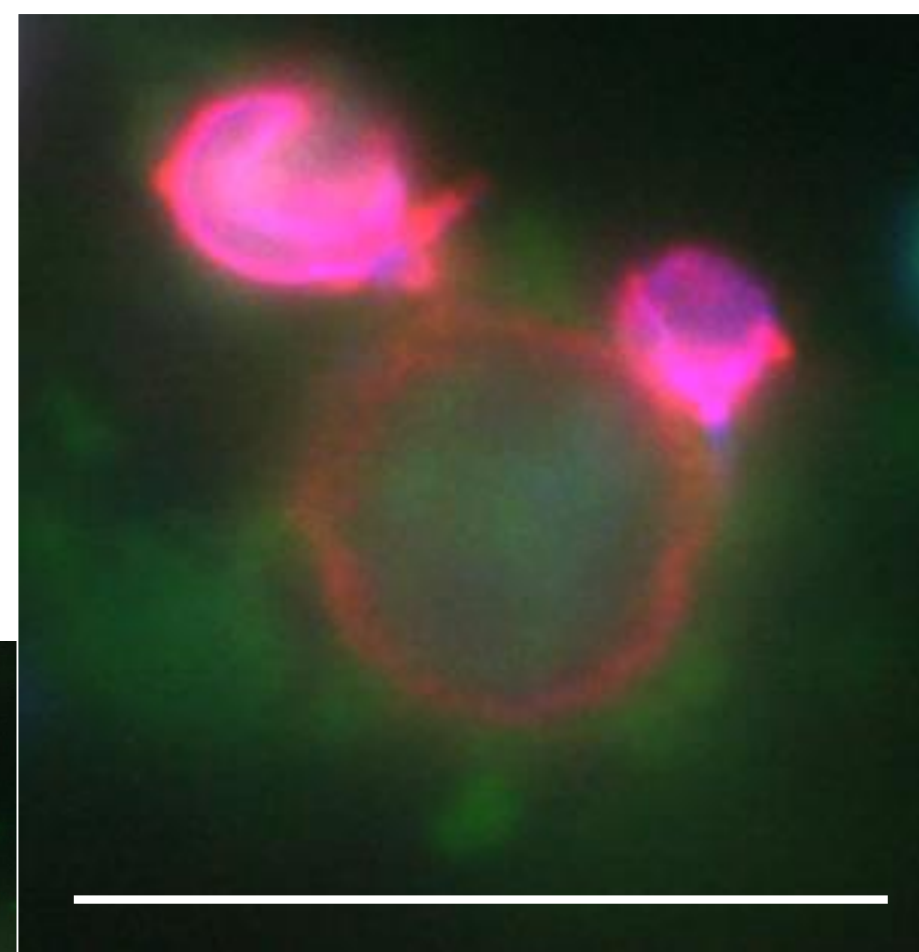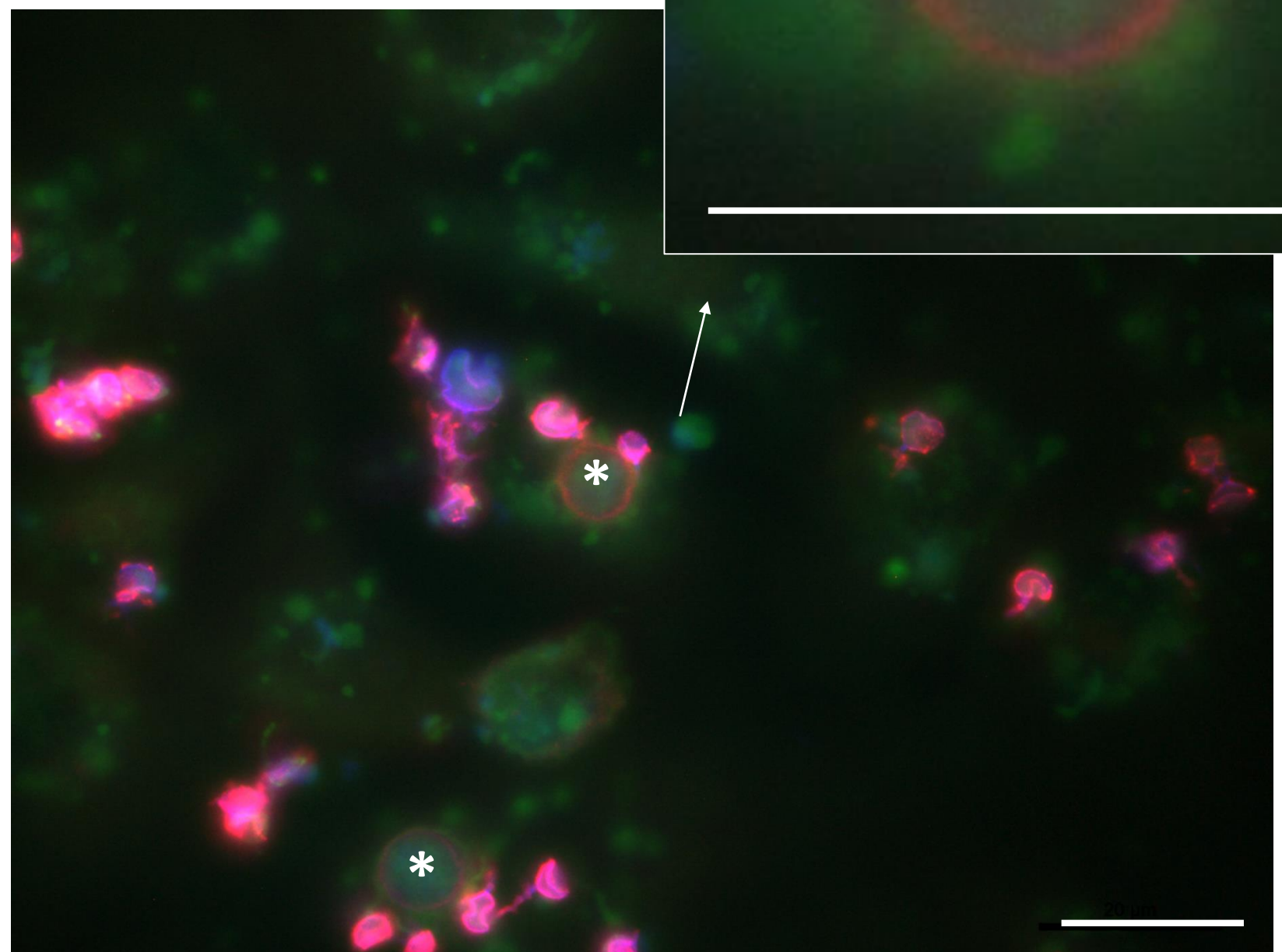

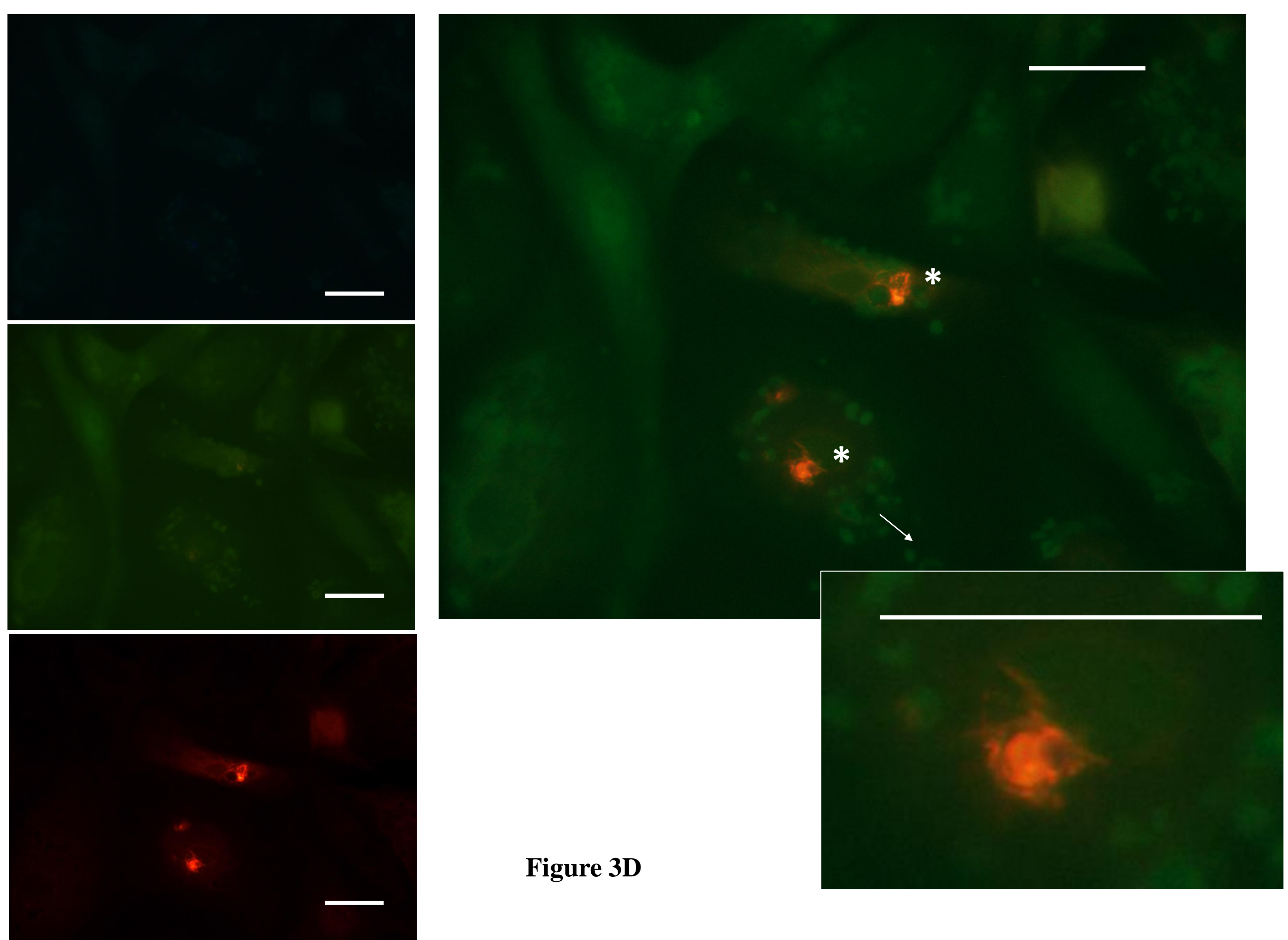

**Figure 3D**

**Figure 3E**

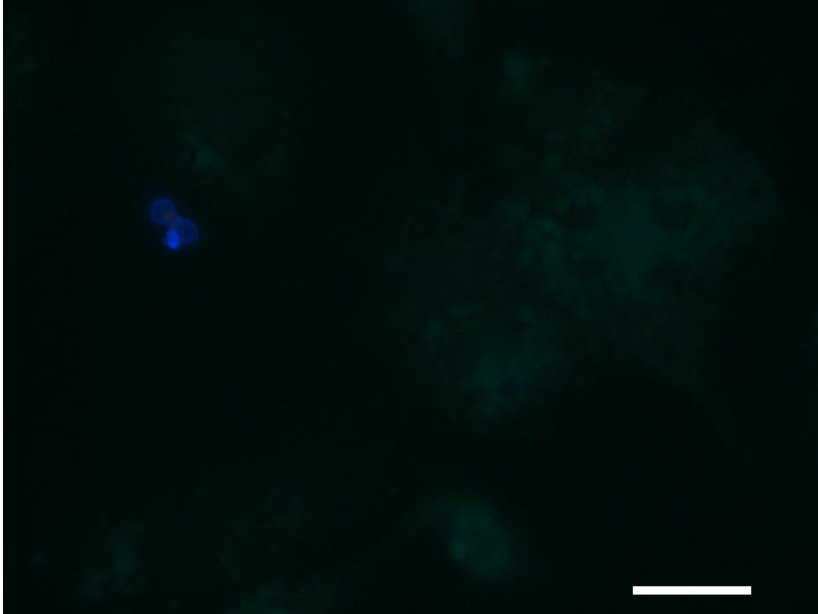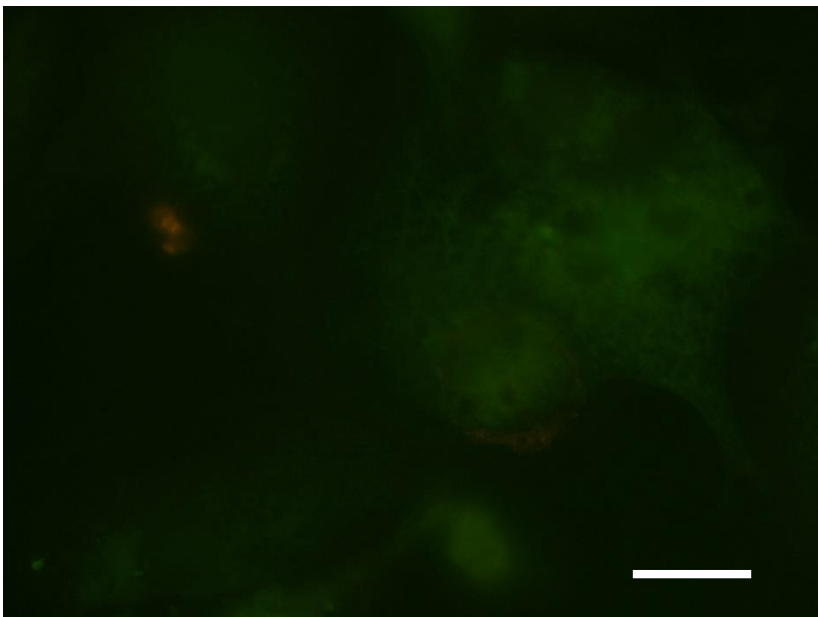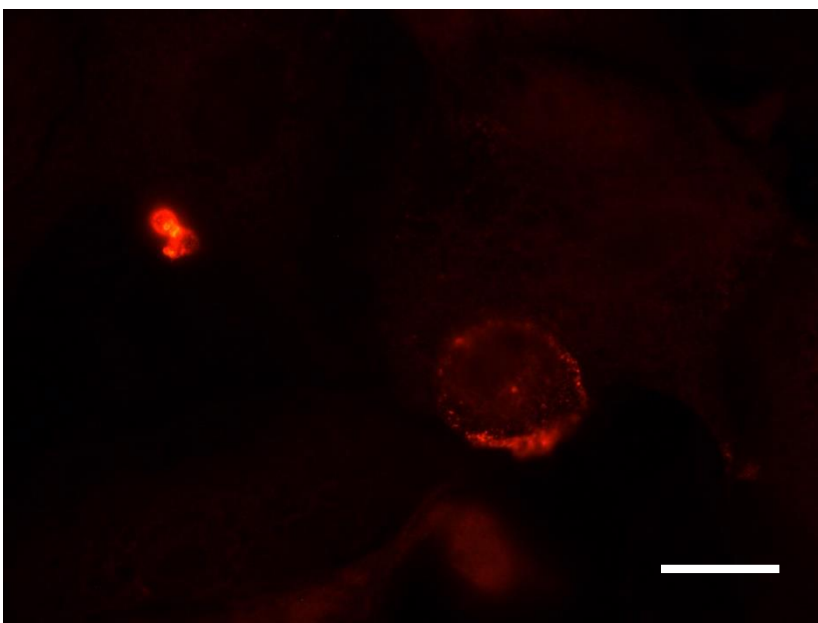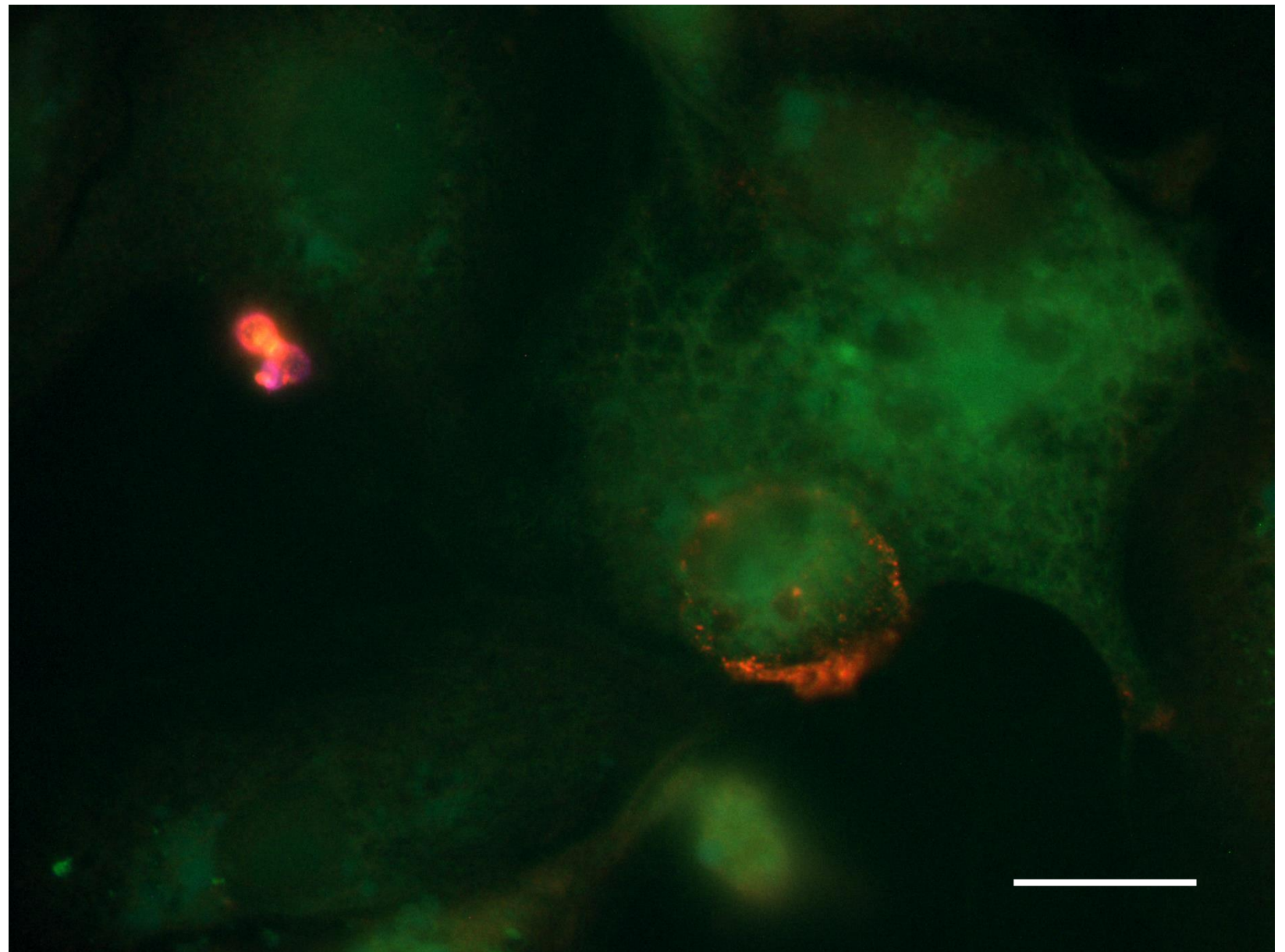

**Figure 3F**

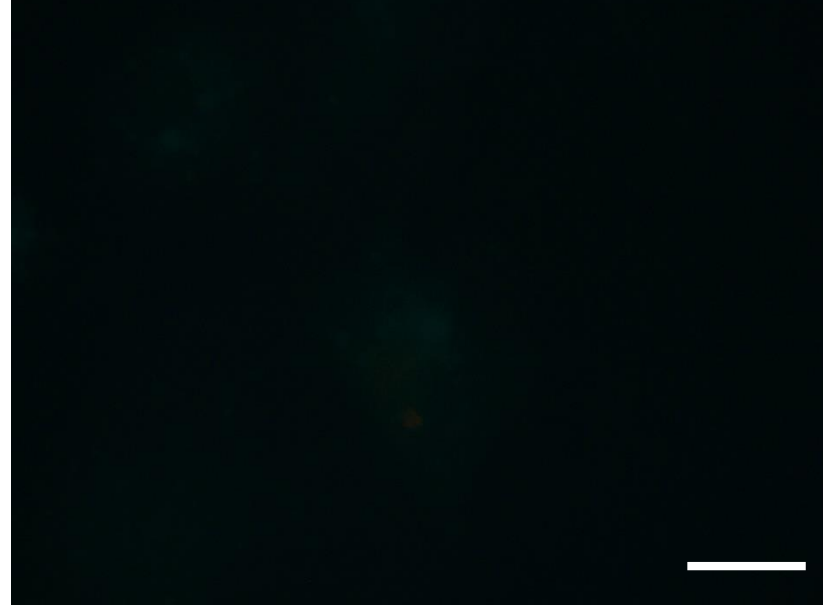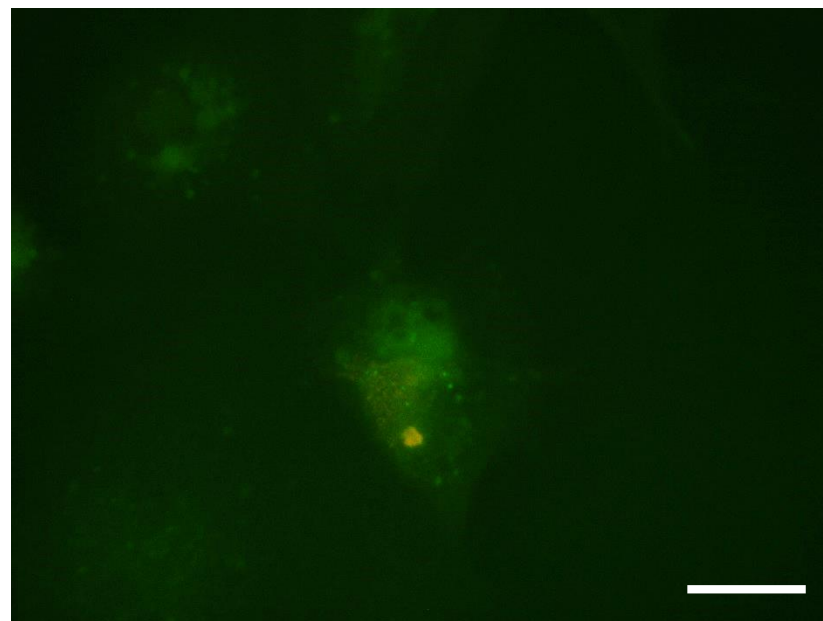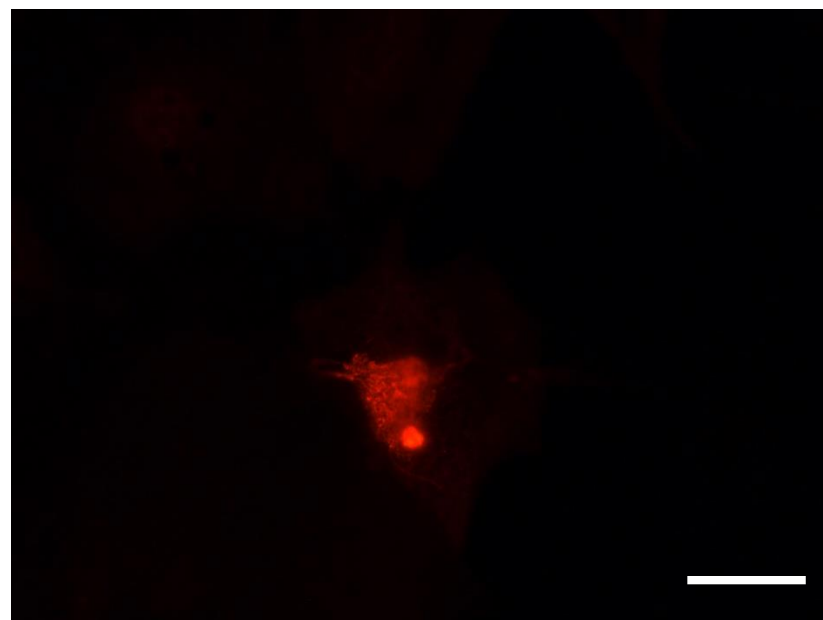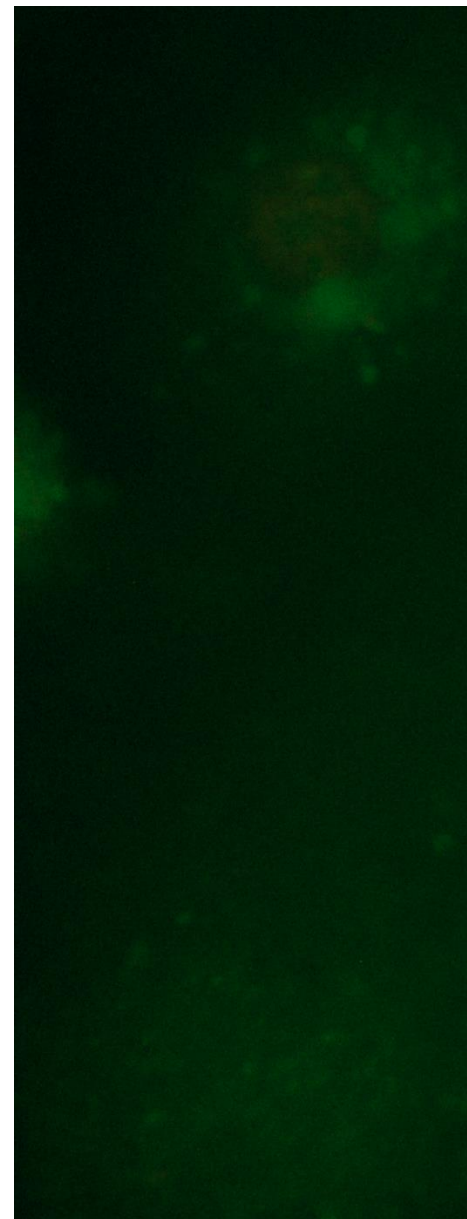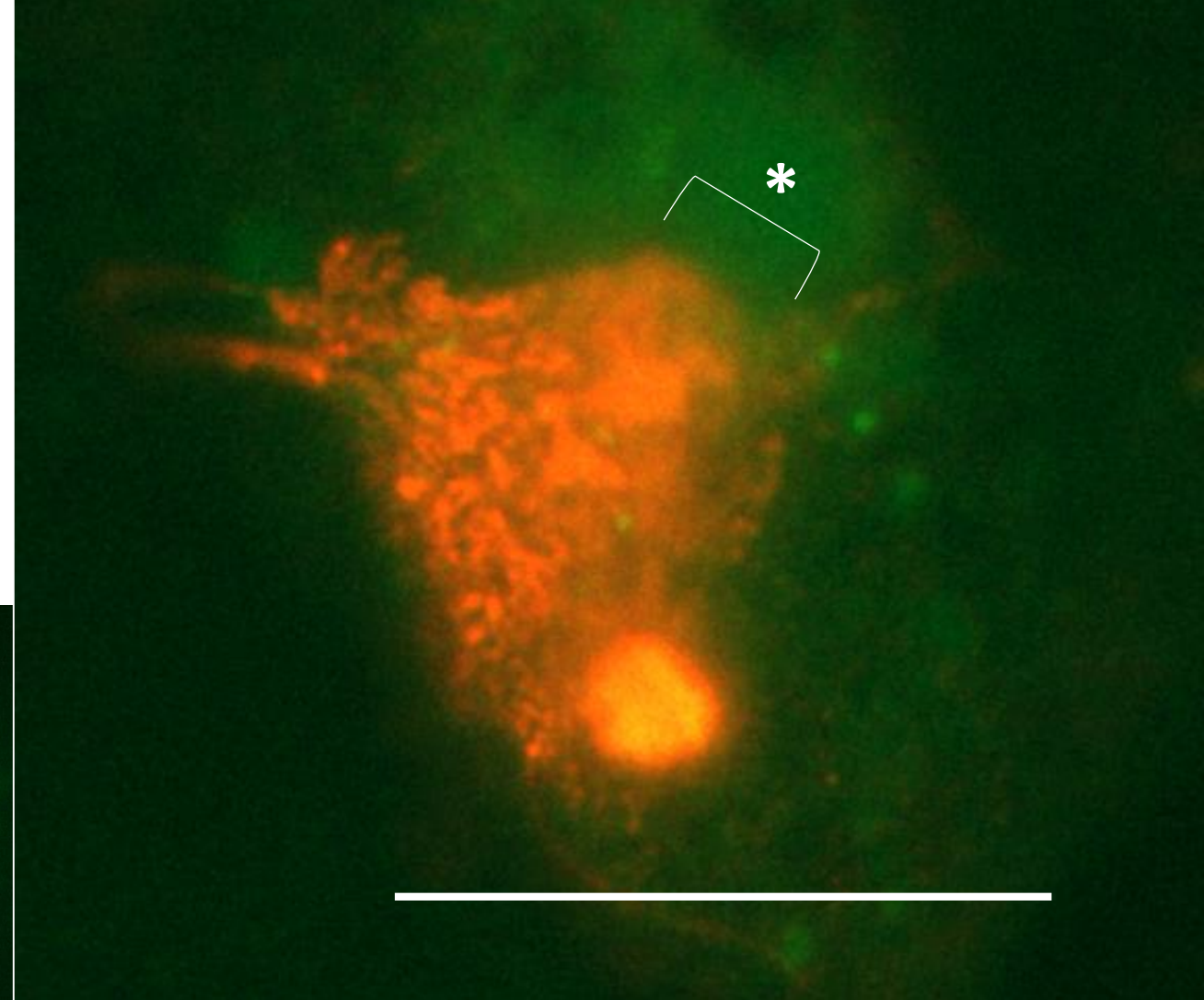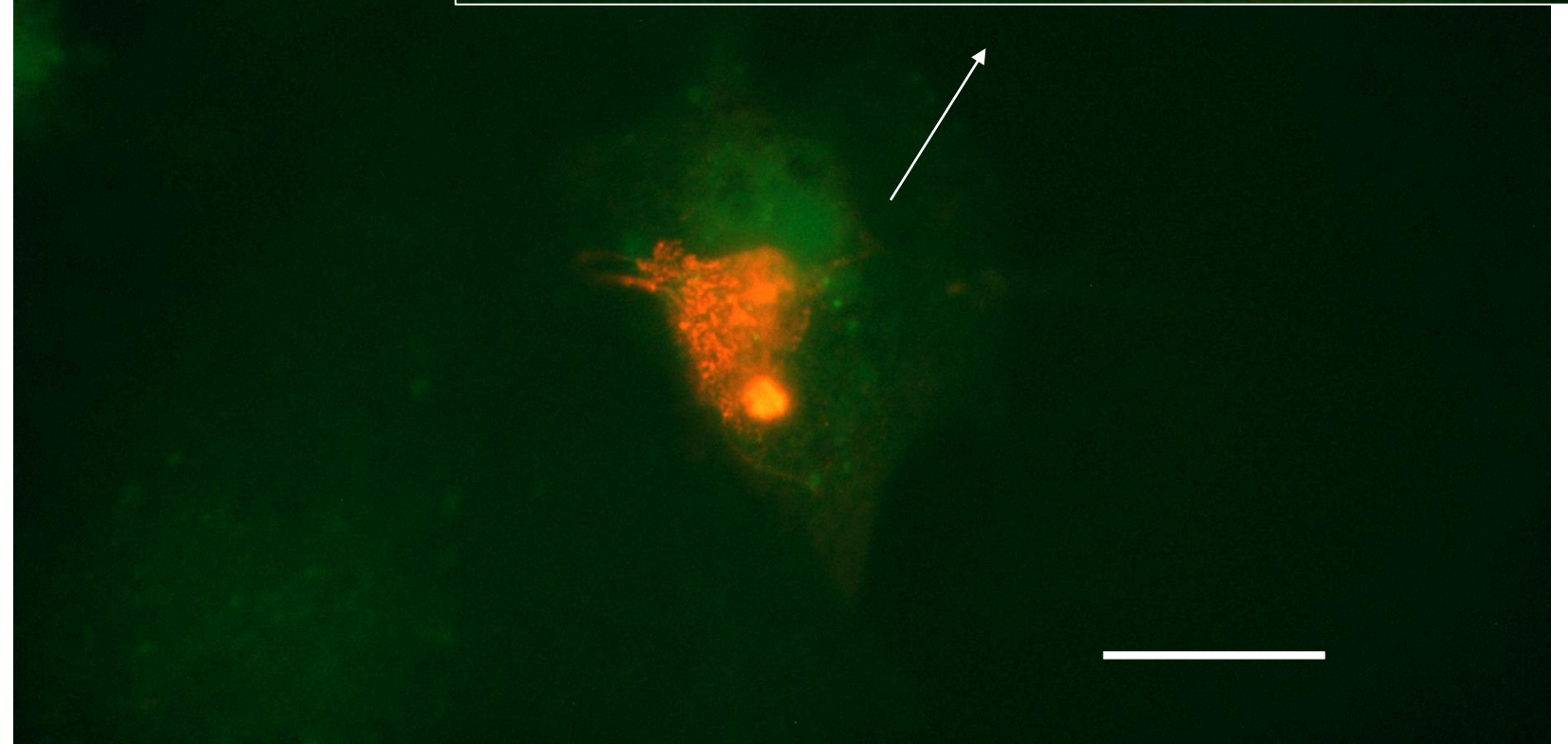

**Figure 3G**

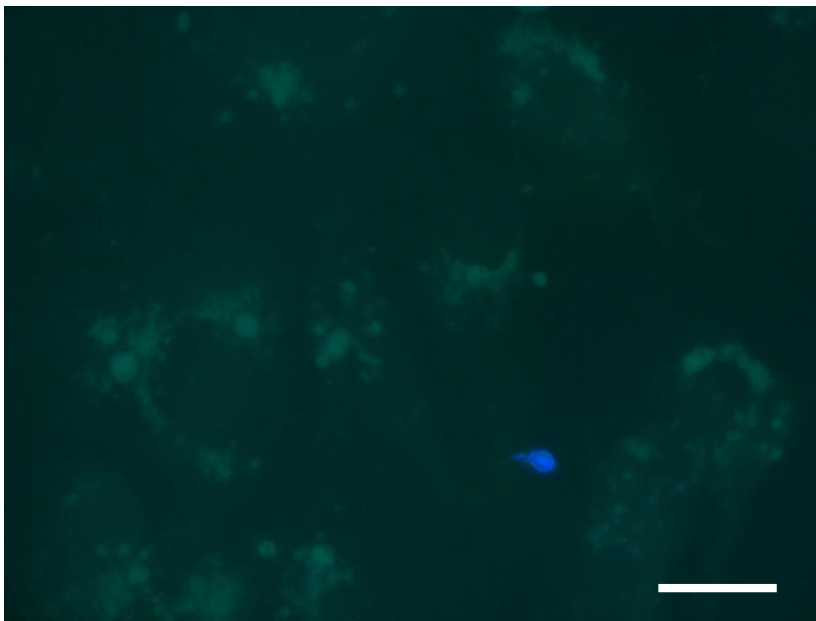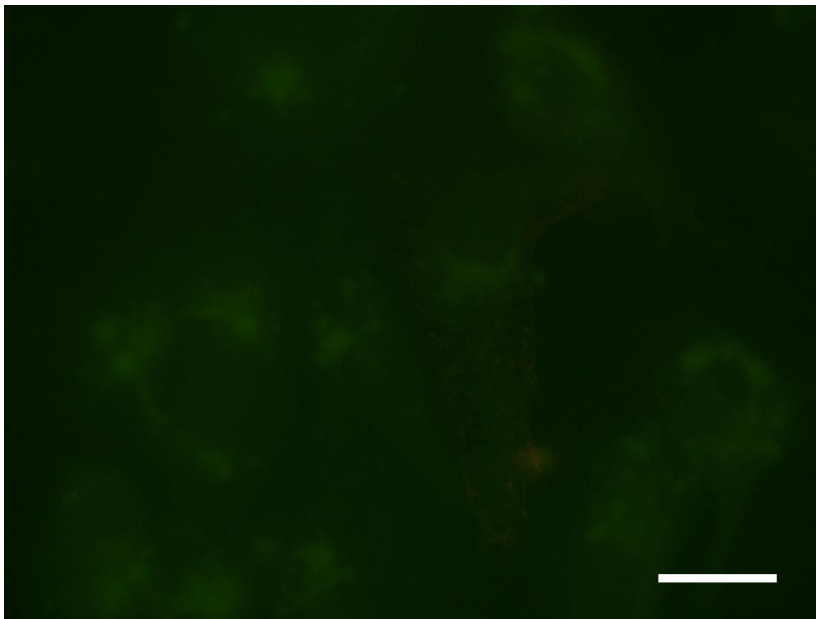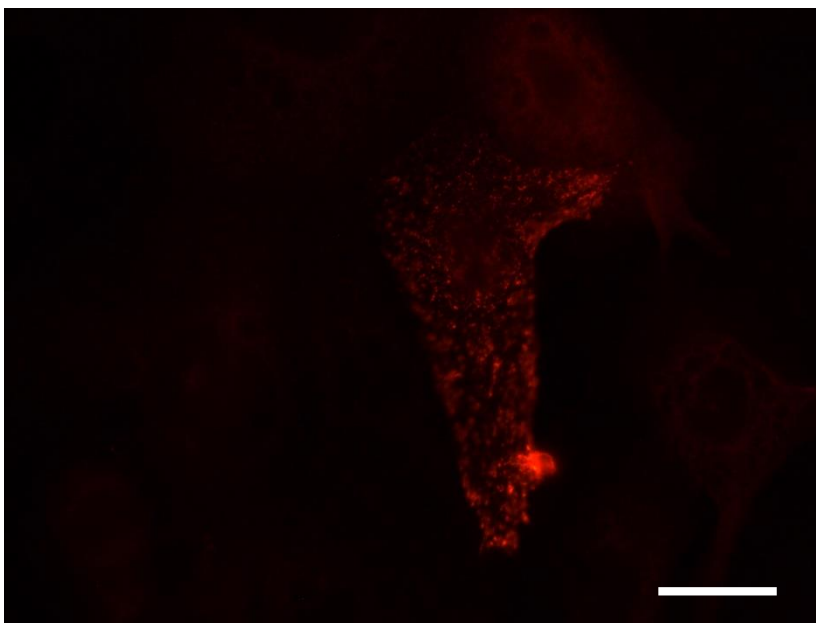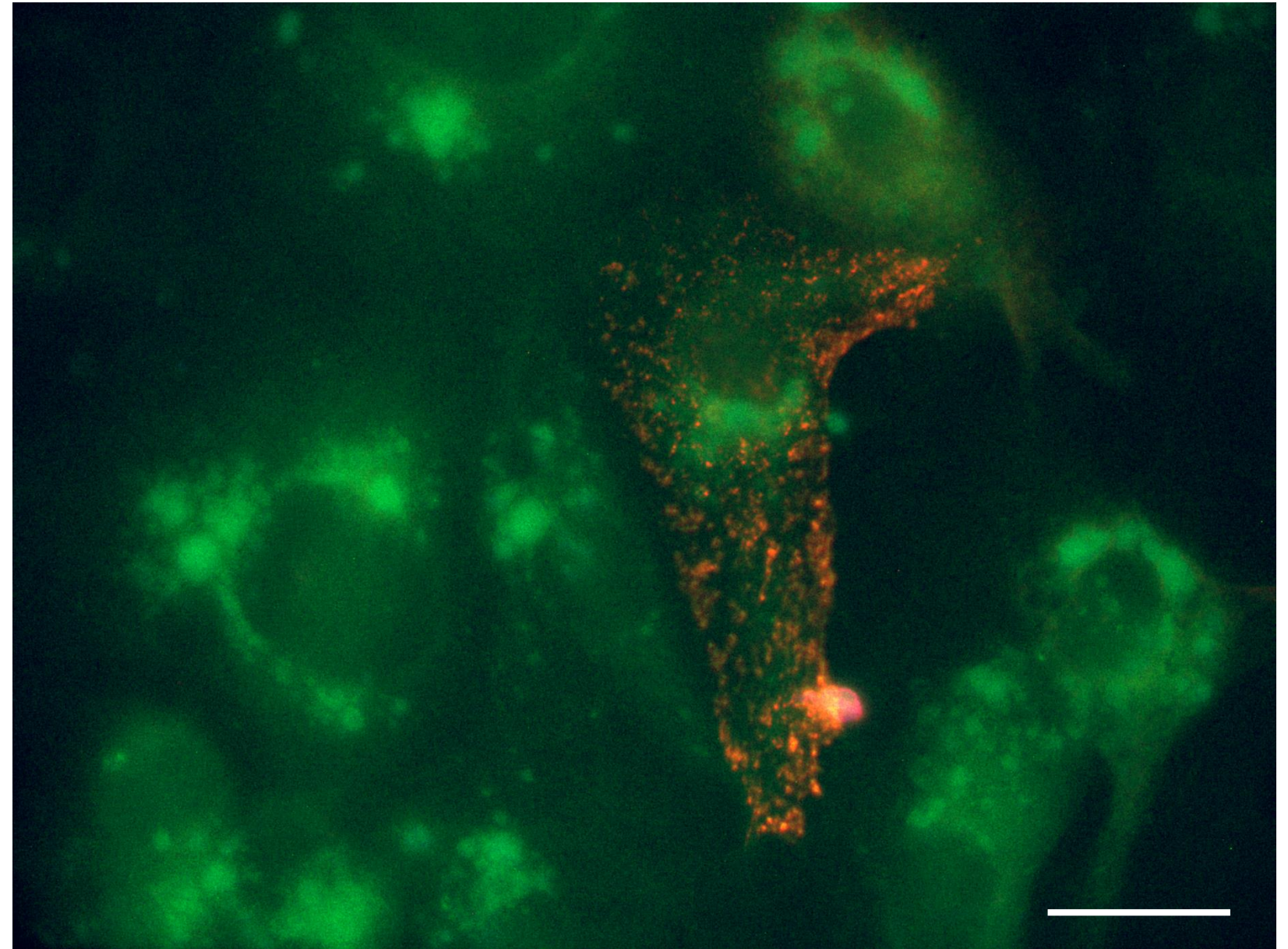

Supplement: S3 File — Shown are the individual fluorescent signals of Bd-infected A6 cells (green cell tracker), extracellular Bd (Calcofluor White (blue)) and extra-and intracellular Bd (Alexa Fluor 568 (red)) and their overlay pictures, which were used in Fig 3. Scale bar = 20 μm. (PDF) [file pone.0225224.s003.pdf]
